# The ABI3 Transcription Factor Interaction and Antagonism with Ubiquitin E3 Ligase ScPRT1 in *Syntrichia caninervis*

**DOI:** 10.3390/genes13050718

**Published:** 2022-04-20

**Authors:** Yigong Zhang, Jiyang Zhou, Yi Zhang, Daoyuan Zhang

**Affiliations:** 1 Xinjiang Key Laboratory of Biological Resources and Genetic Engineering, College of Life Science and Technology, Xinjiang University, Urumqi 830017, China; zhangyg@xju.edu.cn (Y.Z.); zhoujiyang@xju.edu.cn (J.Z.); zhangyi@stu.xju.edu.cn (Y.Z.); 2 Xinjiang Key Laboratory of Conservation and Utilization of Plant Gene Resources, Xinjiang Institute of Ecology and Geography, Chinese Academy of Sciences, Urumqi 830011, China; 3 Turpan Eremophytes Botanical Garden, Chinese Academy of Sciences, Turpan 838099, China

**Keywords:** desiccation-tolerant moss, E3 ubiquitin ligase, ScABI3, ScPRT1, abscisic acid

## Abstract

The ubiquitination pathway has been found to regulate plant responses to environmental stress. However, the role of E3 ubiquitin ligase in desiccation tolerant moss has not yet been elucidated. Previous research has shown that the abscisic acid (ABA) signaling factor ScABI3 can significantly increase desiccation tolerance and reduce ABA sensitivity in the desert moss *Syntrichia caninervis*. In this study, we identified a RING-type E3 ubiquitin ligase, ScPRT1, and showed that ScABI3 can directly interact with ScPRT1 in vitro and in vivo. Furthermore, we found that the high expression of *ScPRT1* can interfere with the transcription of *ScABI3* under ABA treatment. Therefore, we speculate that ScPRT1 may degrade ScABI3 through the ubiquitin-26S proteasome system and participate in ABA-dependent signaling in response to ABA-insensitivity or desiccation tolerance in *S. caninervis*. The findings from our study may enrich our knowledge of the role of E3 ubiquitin ligase in desiccation tolerance and lay a theoretical foundation for an in-depth study of the relationship between ubiquitination modification and ABA signal transduction under environmental stress.

## 1. Introduction

Plants, as sessile organisms, must adapt in situ to changing environmental conditions. Over evolutionary history, plants have evolved a set of adaptive mechanisms to effectively deal with abiotic and biotic stressors. One such mechanism, ubiquitination, a protein post-translational modification, plays an important role in diverse aspects of eukaryotic cell regulation, especially in the plant stress response [1,2]. Ubiquitination provides flexibility and diversity in response to different environmental conditions by modulating protein turnover and homeostasis through the addition of one or more ubiquitins in different configurations [3]. Conjugation occurs via the sequential actions of three enzyme families which ultimately couple ATP hydrolysis to isopeptide bond formation: the E1 ubiquitin-activating enzymes, the E2 ubiquitin-conjugating enzymes, and the E3 ubiquitin-protein ligases [4]. Specifically, E3 ubiquitin ligase plays a critical role in the ubiquitin proteasome system (UPS) by selecting appropriately from a myriad of candidate proteins during ubiquitination, thus regulating the plants’ response to abiotic stress [5,6].

The plant hormone abscisic acid (ABA) can catalyze the binding of ubiquitin to target proteins through E3 ligase, at the same time, E3 ligase can participate in the regulation of ABA stability, and play a key role in ABA signal perception and regulation [7]. ABSCISIC ACID INSENSITIVE 3 (ABI3), a transcription factor found in a variety of plants, is a key regulator of ABA signal transduction. As a regulatory gene, *ABI3* can together with interacting factors, respond to various phytohormone signaling pathways and participate in regulating plant growth and stress response [8,9,10]. Specifically, *ABI3* can bind to the regulatory elements in the promoter region of the target gene through its B1, B2, and B3 domains, and thus regulate the physiological and biochemical processes of plants by activating or inhibiting the transcriptional activity of the target gene [11,12,13]. The relationship between ABI3 and E3 ubiquitin ligase has been studied in many plants. For example, the RING-type E3 ligase AIP2 negatively regulates ABA signaling through the degradation of ABI3 in *Arabidopsis thaliana* [14]. Similarly in wheat, the AIP2 homolog TaAIP2 negatively regulates ABA signaling and controls seed panicle germination by polyubiquitination of TaABI3 [15]. In rice, the AIP2 homologous protein OsDSG1 can interact with and degrade OsABI3 by ubiquitination in order to regulate seed germination and abiotic stress [16]. In addition, ABI3-regulated downstream transcription factor DREB2A is also a target protein of RING-type E3 ubiquitinates DRIP1 and DRIP2, and negatively regulates the water stress response [17]. FUS3, in the same subgroup of ABI3, is also regulated by AIP2 ubiquitination and plays a role in cotyledon development and flowering in *A. thaliana* [18].

*S. caninervis*, a desert moss that is naturally exposed to unpredictable cycles of dehydration and rehydration, can tolerate severe desiccation. Amazingly, *S. caninervis* can lose up to 90% of its tissue water, and upon rehydration, recover and restore normal growth within 30 s [19,20]. This trait makes *S. caninervis* an ideal model for elucidating the molecular mechanisms of desiccation tolerance in non-vascular plants [21]. In our previous work, we cloned the *ABI3* gene from *S. caninervis*, and found that *ScABI3* transgenic *A. thaliana* had improved salt and drought tolerance, increased expression of stress-related genes, and reduced ABA sensitivity [22]. We performed yeast two-hybrid screening using the *S. caninervis* cDNA library as prey and ScABI3 as bait to investigate the interaction between ScABI3 and other proteins, the results showed that ScABI3 can interact with multiple proteins, including an E3 ubiquitin ligase.

Previous research has reported that ubiquitination modification modulates the ABA signaling pathway in vascular plants [23,24,25]. However, the specific role of E3 ubiquitin ligase in desiccation-tolerant moss is still unclear, and precisely how the ubiquitination modification modulates the ABI3 transcription factor is unexplored. Here, we isolated a putative C3HC4-RING ubiquitin E3 ligase PRT1 (PROTEOLYSIS1) from *S. caninervis*, and revealed that ScPRT1 physically interacts with ScABI3 in vitro and in vivo. Moreover, we showed that overexpression of *ScABI3* significantly increased the cotyledon emergence rates by over 80% compared with WT, and *ScPRT1* antagonism against *ScABI3* transcription during ABA treatment. This study provides new insights into the modulation mechanism of ubiquitination modification on the ABA signaling pathway in desiccation-tolerant moss.

## 2. Materials and Methods

### 2.1. Gene Cloning and Bioinformatics Analysis

The *PRT1* gene was cloned by the RT-PCR method from the genome of *S. caninervis* [21]. Multiple sequence alignments and phylogenetic trees were constructed using the DNAMAN software ver. 5.2.2.0 (Lynnon BioSoft, San Ramon, CA, USA) and the BLASTN program (NCBI, Bethesda, MD, USA).

### 2.2. Analysis of ScABI3 Transgenic A. thaliana Germination under ABA Treatment

*ScABI3* transgenic *A. thaliana* plants were obtained as described by Zhang et al. [22]. Seeds of wild-type Columbia 0 (WT) and three independent transgenic lines (line 1, line 2, and line 3) were surface sterilized and sown on MS agar medium (controls) or MS agar medium supplemented with 0.5, 1.0, or 2.0 µM ABA as treatments. Cotyledon emergence rates were scored after 7 days of 16/8 h light/dark cycles at 23 °C with a light intensity of 100 µmol photons m^−2^ s^−1^. All experiments have been replicated a minimum of three times.

### 2.3. Yeast Two-Hybrid Assay

The truncated sequence of *ScABI3* was used to construct the pGBKT7-*ScABI3* yeast expression vector as bait because the full-length sequence has an autoactivation domain [22]. *ScPRT1* which was verified to have no autoactivation was used to construct the pGADT7-*ScPRT1* yeast expression vector, as prey protein. A mixture of both vectors was transformed into the yeast strain AH109 by the lithium acetate method following the Yeast Protocols Handbook (Clontech Laboratories, Mountain View, CA, USA), plated on the SD/-Trp-Leu or SD/-Trp-Leu-His selection medium and incubated at 28 °C for 3 days to identify the transformants. We selected colonies from SD/-Trp-Leu-His and plated them on SD/-Trp-Leu-His-Ade medium again for further selection. The interaction was confirmed by plating on SD/-Trp-Leu-His-Ade medium containing X-α-gal. The combination of pGBKT7-p53+pGADT7-largeT was used as a positive control and the combination of pGBKT7-laminC+pGADT7-largeT empty vector was used as a negative control. Three independent experiments were performed and similar results were obtained.

### 2.4. Pull-Down Assays

The coding sequence (CDS) of *ScABI3* was cloned into the pD2P vector with a GFP tag. The CDS of *ScPRT1* was cloned into the pD2P vector fused with a His-tag. The recombinant proteins were expressed in cell-free protein expression according to the protocol of Protein Factory 1.0 (Kangma-Healthcode, Shanghai, China). GFP and GFP-tagged ScABI3 were purified by GFP-trap beads according to the manufacturer’s protocol (GE Healthcare, Marlborough, MA, USA). His and His-tagged ScPRT1 protein were purified by Ni-NTA beads (Qiagen, Hilden, Germany). Subsequently, 5 μg samples of ScABI3-GFP or GFP were incubated with 5 μg samples of ScPRT1-His in 500 μL pull-down buffer (150 mM NaCl, 20 mM Tris, 1 mM phenyl-methylsulfonyl fluoride, 0.2% Triton X-100, 1% protease inhibitor cocktail, pH 8.0) at 4 °C for 2 h. The beads were washed thoroughly with the pull-down buffer and proteins were eluted from the beads by boiling at 95 °C with 30 μL sodium dodecyl sulfate-polyacrylamide gel electrophoresis (SDS-PAGE) loading buffer for 10 min and then separated by SDS-PAGE. Immunoblot assays were performed using the anti-His (Abmart, Berkeley Heights, NJ, USA) and anti-GFP (Abmart, Berkeley Heights, NJ, USA) antibodies, respectively.

### 2.5. Bimolecular Fluorescence Complementation (BiFC) Assays

The BiFC vector was designed based on the split fluorescent protein (YFP). Full-length coding cDNA sequences of *ScABI3* and *ScPRT1* were cloned into the pCAMBIA1300-35S-YC155 and pCAMBIA1300-35S-NY173 vectors, respectively, which were then transformed into *Agrobacterium tumefaciens* strain GV3101. The strain was incubated in LB medium overnight and resuspended in infiltration buffer (10 mM MgCl_2_, 10 mM MES and 100 µM acetosyringone) to a final concentration of OD_600_ = 0.4 and placed at room temperature (25 °C) for 2–3 h before infiltration into *Nicotiana benthamiana* leaves. Infiltration was achieved by forcing a solution of suspended *A.tumefaciens* cells into leaves, while still attached to the plant, by applying positive pressure with a syringe as described by Kudla and Bock [26]. Plants with infiltrated leaves were incubated in the greenhouse for 3 days under 25 °C, 150 µmol photons m^−2^ s^−1^, and RH = 25%. The YFP fluorescent signal in the leaf was examined under a confocal microscope (LSM700, Carl Zeiss, Oberkochen, Germany) with 514 nm excitation. Three replicates were applied to each treatment.

### 2.6. Expression Analysis of ScABI3 and ScPRT1 Genes under Different Abiotic Stress Treatments in S. caninervis

To evaluate the expression levels of *ScABI3* and *ScPRT1*, 10–12 gametophores were collected at different time points, for a total of 50–60 dry gametophore samples. For ABA treatments, dry samples were fully hydrated for 24 h, transferred to new Petri plates containing filter papers saturated with 100 µM ABA, and incubated for 0, 0.5, 1, 2, 4, 6, 8, 12, and 24 h at room temperature (25 °C, 150 µmol photons m^−2^ s^−1^, and RH = 25%). For dehydration treatments, dry gametophores were fully hydrated for 24 h and air-dried for the same processing times as the ABA treatments at room temperature. For rehydration treatments, dry gametophores were transferred to Petri plates containing filter papers saturated with 8 mL of filtered water and incubated for the same processing times as the ABA treatments, at room temperature. For salt stress, dry samples were fully hydrated for 24 h, transferred to new Petri plates containing filter papers saturated with 250 µM NaCl, and incubated for the same processing times as the ABA treatments, at room temperature.

All harvested samples were flash-frozen in liquid N_2_ and stored at −80 °C prior to RNA extraction. Total RNA was extracted using E.Z.N.A. Plant RNA Kit (Omega Bio-Tek, Norcross, GA, USA). cDNA was synthesized using random hexamer primers with PrimeScript RT reagent Kit (Takara Bio, Shiga, Japan). The qRT-PCR primers were designed using the Primer Premier 5.0 software (Premier Biosoft, San Francisco, CA, USA) (Table 1). qRT-PCR was performed using the SYBR Premix Ex Taq II (TIi RNaseH Plus, Takara, Bio, Shiga, Japan). The fluorescence intensity was measured with a CFX96 Real-Time System (Bio-Rad, Hercules, CA, USA). The target gene expression levels were normalized using the *α-tubulin2* gene as an internal reference [27]. The relative abundance of the transcripts compared to the reference gene was calculated according to the 2^−ΔΔCt^ method [28]. Each reaction was performed in triplicate.

### 2.7. Statistical Analysis

Statistical analyses were performed using SPSS version 18.0 (SPSS Inc., Chicago, IL, USA). Data were compared by one-way analysis of variance (ANOVA), and differences were considered statistically significant at *p* < 0.05 and substantially significant at *p* < 0.01.

## 3. Results

### 3.1. Multiple Sequence Alignment and Phylogenetic Analysis of ScPRT1

**Figure 1:**
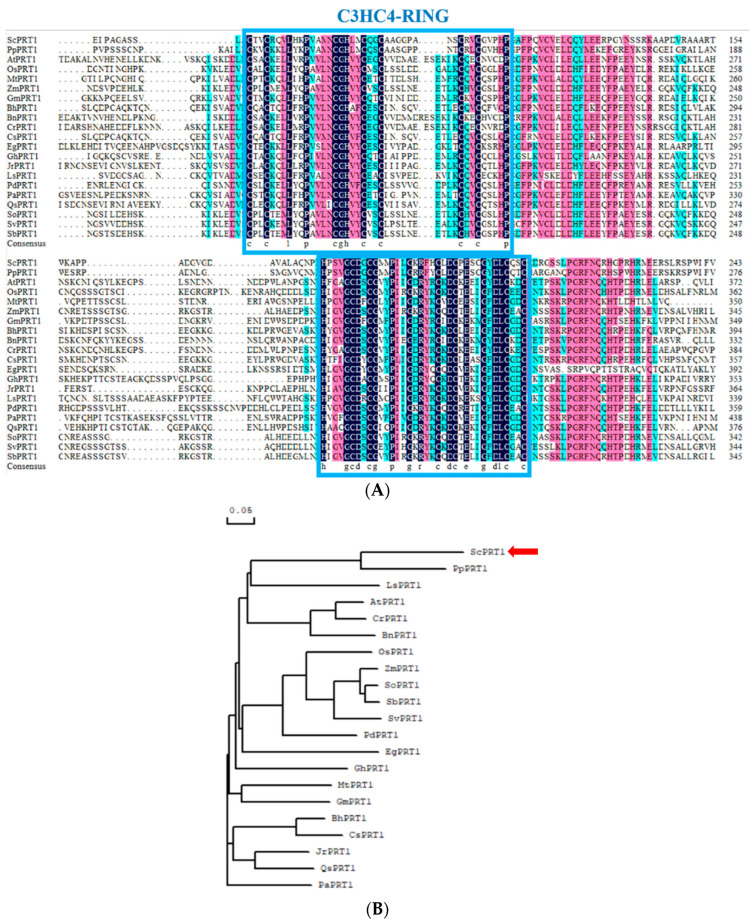
Multiple sequence alignment and phylogenetic analysis of ScPRT1. (**A**) Amino acid sequence alignment of ScPRT1 and closely related PRT1 from other plant species. ScPRT1 (*S. caninervis*), PpPRT1 (*P. patens*, XM_024525392.1), AtPRT1 (*A. thaliana*, AY080799.1), OsPRT1 (*Oryza sativa*, XM_015767241.2), MtPRT1 (*Medicago truncatula*, XM_003604918.4), ZmPRT1 (*Zea mays*, XM_020538608.2), GmPRT1 (*Glycine max*, XM_006602468.4), BhPRT1 (*Benincasa hispida*, XM_039021813.1), CrPRT1 (*Capsella rubella*, XM_006297601.2), CsPRT1 (*Cucumis sativus*, XM_004139587.3), EgPRT1 (*Eucalyptus grandis*, XM_010038525.3), GhPRT1 (*Gossypium hirsutum*, XM_016840815.2), JrPRT1 (*Juglans regia*, XM_018985173.2), LsPRT1 (*Lactuca sativa*, XM_023899275.2), PdPRT1 (*Phoenix dactylifera*, XM_008786960.4), PaPRT1 (*Populus alba*, XM_035060044.1), QsPRT1 (*Quercus suber*, XM_024035664.1), SoPRT1 (*Saccharum officinarum*, MT747433.1), SvPRT1 (*Setaria viridis*, XM_034746023.1), SbPRT1 (*Sorghum bicolor*, XM_002454141.2). Multiple sequence alignments were conducted using the DNAMAN software. Common identical amino acid residues are shown with a dark background and similar residues are shown with a light background. The C3HC4-RING domains are indicated by the blue box. (**B**) Phylogenetic relationships between ScPRT1 and other reported PRT1 from *P. patens* and higher plants. The phylogenetic tree was constructed using the neighbor-joining method in MEGA 5.0.

We obtained the genomic sequence of the ScPRT1 protein from the genome of the desiccation-tolerant moss *S. caninervis*. This sequence contains a 711 bp open-reading frame, encoding a protein containing 236 amino acid residues. Multiple sequence alignments of the ScPRT1 ORF with PRT1 ORF of 20 species from NCBI indicate that the ScPRT1 belongs to the C3HC4-RING ubiquitin E3 ligase PRT1 and contains the highly conserved cysteine and histidine functional domain regions (Figure 1A). Phylogenetic analysis indicated that PRT1 proteins form two groups based on overall protein structure and that ScPRT1 clustered in the same branch as the *Physcomitrella patens* PRT1 which indicated a similar ancestry or function (Figure 1B).

### 3.2. Overexpression of ScABI3 Reduced the Sensitivity of Seeds under ABA Treatment

**Figure 2:**
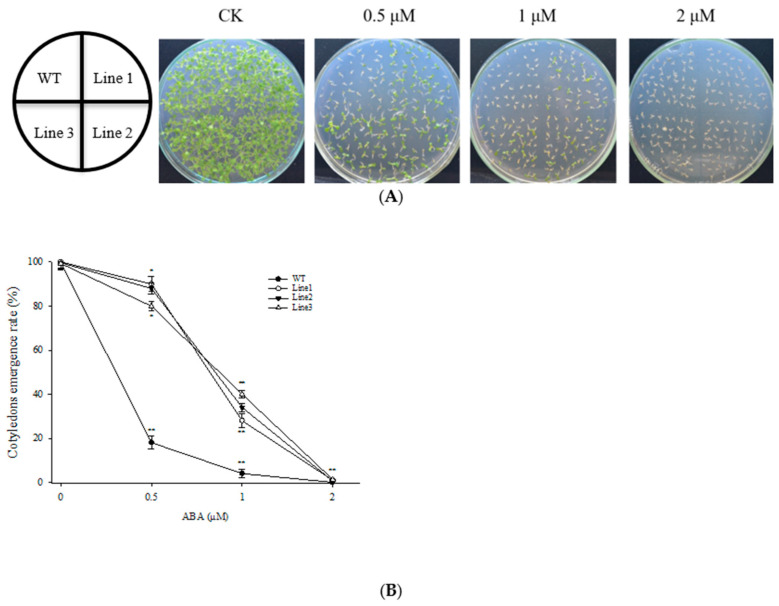
Germination assay of WT and transgenic *A. thaliana* under ABA treatment. (**A**) The germination of four lines on MS containing 0, 0.5, 1.0, and 2.0 µM ABA. (**B**) The cotyledon emergence rate according to A. The experiments have been repeated at least three times. * Indicates a significant difference (*p* < 0.05), and ** indicates a substantially significant difference between the transgenic line and WT (*p* < 0.01).

Three independent transgenic overexpression lines (T3) with the highest accumulation of *ScABI3* (Line 1, Line 2, and Line 3) were chosen for seed germination assays (Figure 2A). The cotyledon emergence rates of WT and transgenic lines were not significantly different in the control MS medium. In contrast, cotyledon emergence rates were severely inhibited for WT seeds by both 0.5 and 1 µM ABA, approximately 18% and 4%, respectively, but were only slightly inhibited in the three transgenic lines, from 80 to 90% and 28 to 40%, respectively, for the 0.5 and 1 µM ABA treatments (Figure 2B).

### 3.3. ScABI3 Interacts with ScPRT1 in Yeast Two-Hybrid Assays

**Figure 3:**
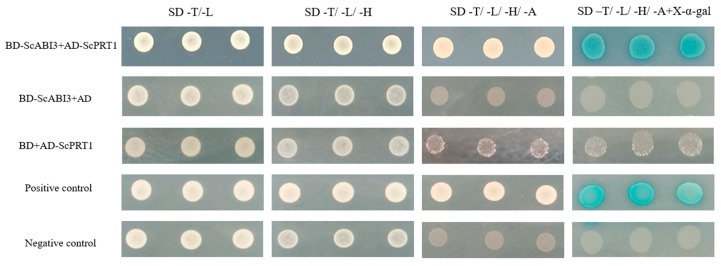
Interaction test by Y2H assay. The transformed cells were plated onto SD-T: -trp, SD-L: -leu; SD-TL: -trp, -leu; SD-TLH: -trp, -leu, -his; SD-TLHA: -trp, -leu, -his, -ade medium. The known pGBKT7-p53 + pGADT7-largeT vector was used as a positive control, and the pGBKT7-laminC + pGADT7-largeT vector was used as a negative control.

We isolated ScABI3-interacting protein candidate ScPRT1 for further analysis and performed a one-by-one Y2H assay to confirm the interaction between ScABI3 and ScPRT1. The results revealed that ScABI3 could strongly interact with ScPRT1 in yeast (Figure 3).

### 3.4. ScABI3 Interaction with ScPRT1 In Vitro by Pull-Down Assays

**Figure 4:**
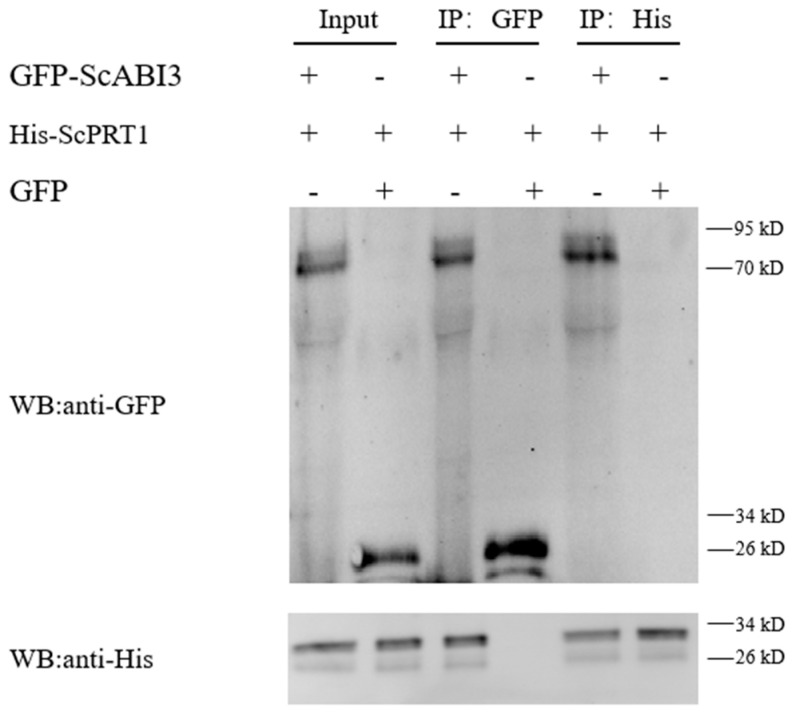
Pull-down assay shows that ScABI3 directly interacts with ScPRT1 in vitro. His-ScPRT1 was pulled down by GFP-ScABI3 immobilized on anti-GFP affinity beads or Ni-NTA magnetic agarose beads and analyzed by Western blotting using an anti-GFP or anti-His antibody. GFP-ScABI3 protein was incubated with protein extracts containing His-ScPRT1 and further immobilized with anti-GFP and anti-His antibodies.

In this study, His-tagged ScPRT1 was immobilized on the Ni column and assayed for the ability to pull down the GFP-ScABI3 fusion protein. Pulldown results were analyzed by immunoblotting with anti-GFP and anti-His antibodies. As shown in the first lane of Figure 3, which shows the pulldown result compared to GFP only (lane 2), purified GFP-ScABI3 (lane 3), and purified His-ScPRT1 (lane 5), both His-ScPRT1 and GFP-ScABI3 bands were simultaneously present in the membrane. Further Western blot analysis detected another protein, which suggests that His-ScPRT1 can interact with GFP-ScABI3 directly in vitro (Figure 4).

### 3.5. ScABI3 Interaction with ScPRT1 by BiFC in N. benthamiana

**Figure 5:**
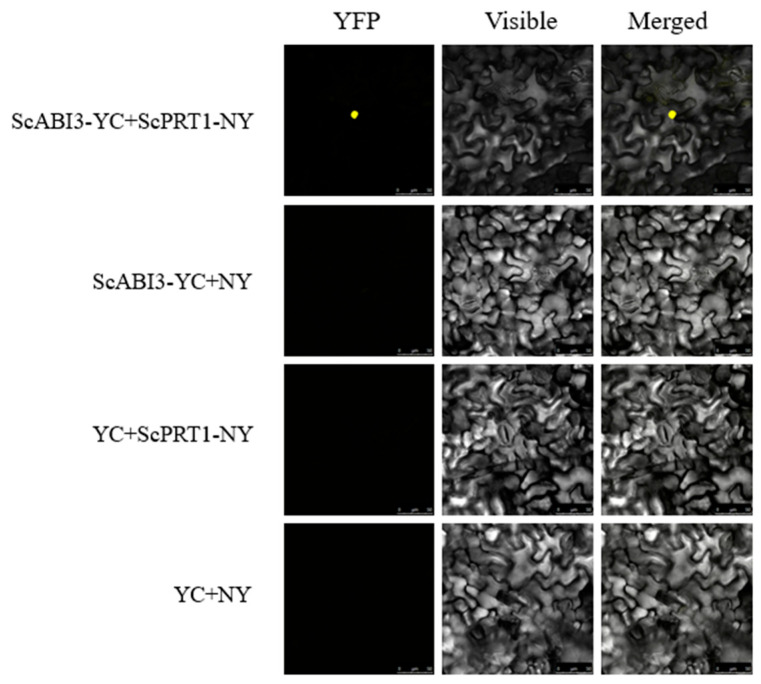
BiFC assay shows that ScABI3 interacts with ScPRT1 in *N. benthamiana* leaf epidermal cells. ScABI3 was fused to the C-terminal fragment of YFP (YC) and ScPRT1 was fused to the N-terminal fragment of YFP (NY). The interaction between ScABI3-YC and NY or YC and ScPRT1-NY or YC and NY serves as the negative controls.

To further characterize the interaction between ScABI3 and ScPRT1 in vivo, a BiFC assay was performed by co-infiltration of recombinant strain combination 35S::ScABI3-YC + 35S::ScPRT1-NY into *N. benthamiana* leaves to observe the fluorescence. As a result, a strong yellow fluorescent signal was observed on the nuclei of the epidermal cells when either of the above combinations was delivered into the tobacco plant, compared to no fluorescent signal in cells with any of the other combinations (Figure 5). This result verified that ScABI3 potentially interacted with ScPRT1 on the nuclei.

### 3.6. ScABI3 and ScPRT1 Expression Induced by Multiple Abiotic Stresses

**Figure 6:**
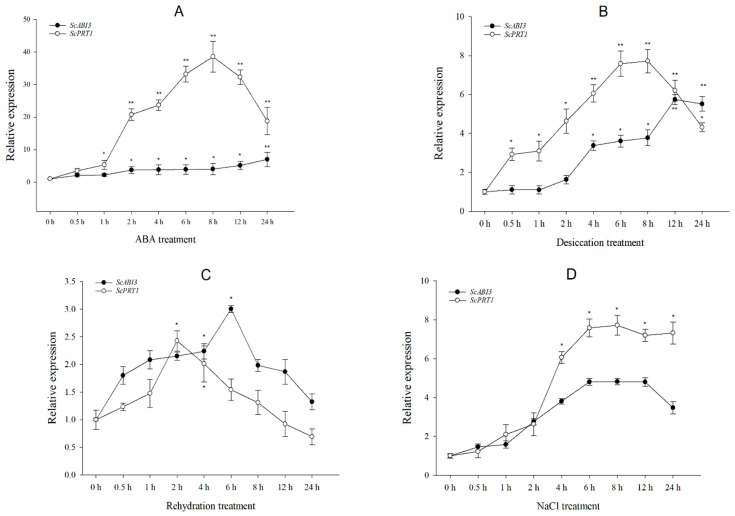
Expression patterns of *ScABI3* and *ScPRT1* in response to different stress treatments. (**A**) ABA, (**B**) dehydration, (**C**) rehydration, (**D**) NaCl treatment. The 2^−ΔΔCT^ method was used in the qRT-PCR analysis, with *α-tubulin* as the internal reference gene and 0 h treatment as the control. Values are means ± SE of three replicates. * Indicates a significant difference (*p* < 0.05). ** Indicates a substantially significant difference (*p* < 0.01).

Transcriptional expression of *ScABI3* and *ScPRT1* was analyzed after exposure to ABA, dehydration, rehydration, and salt treatment (Figure 6). Under ABA treatment, *ScABI3* showed a modest upregulation with increasing time, with the highest expression level at 24 h (Figure 6A), showing only a 6-fold induction compared to 0 h. In contrast, *ScPRT1* was significantly upregulated after 1 h and exhibited an almost 40-fold increase after 8 h of treatment followed by a gradual decline from 12 h to 24 h (but was still significantly higher than at 0 h). In response to desiccation treatment, the transcriptional level of *ScABI3* was significantly induced after 4 h and increased to the highest value from 12 h to 24 h. The accumulation of *ScPRT1* was significantly induced from 0.5 h, reaching a maximal value of more than seven times the value from 6 h to 8 h. Overall, the transcript abundance of *ScPRT1* was higher at each point under desiccation tolerance, except for 24 h (Figure 6B). Under rehydration treatment, the transcripts of *ScABI3* and *ScPRT1* both showed significant accumulation at the beginning and then decreased with increasing time. *ScABI3* abundance reached a maximal value of 3.0 times that of the control at 6 h. In contrast, the transcript abundance of *ScPRT1* reached its maximal value of 2.4 times that of the control at 2 h (Figure 6C). Under NaCl treatment, the transcript abundance of *ScABI3* had a 4.8-fold increase during 6–12 h of treatment, compared to the control condition. Meanwhile, the highest expression level of *ScPRT1* was observed around 4–24 h with a greater than 6.0-fold increase compared with 0 h (Figure 6D).

## 4. Discussion

The plant hormone ABA can activate diverse adaptive responses to unfavorable environments and is especially vital for desiccation tolerance acquisition [23,29]. The E3 ligases can interact with and degrade specific substrates by ubiquitination and are also involved in ABA signal transduction by diverse means [30]. For example, The RING-type E3 ligase SDIR1 is located upstream of ABI5, ABF3, and ABF4, which are positive regulators in ABA signal transduction. SDIR1 selectively regulates the expression of the downstream transcription factor ABI5 by affecting the stability of its substrate SDIRIP1, thereby regulating ABA-mediated seed germination and salt stress [6]. RING-type E3 ligases RGLG5 and RGLG1 release the blockade of ABA signaling by PP2C by regulating the degradation of PP2C protein and activating the ABA signaling pathway [31]. The SCF-type E3 ligase complex AtPP2-B11 can directly interact with and degrade SnRK2.3, negatively regulating the plant response to ABA [32]. U-box E3 ligases PUB12 and PUB13 can also interact with ABI1. When ABA and PYR1 are present at the same time, PUB12 and PUB13 can ubiquitinate ABI1 and participate in plant dehydration [33]. Plants overexpressing U-box E3 ligase PUB10 showed the phenotype of *myc2* mutant, and under ABA treatment, PUB10 plants showed the phenotype of MYC2 overexpression, which indicated that PUB10 can degrade MYC2 through ubiquitination and negatively regulate ABA signaling [34].

E3 ligases modulate the activity and localization of key factors in the ABA signaling pathway by ubiquitination and thus affect the response of plants to ABA. Although a number of E3 ligases with positive and negative regulatory functions involved in the regulation of ABA signaling have been found in vascular plants, research on ubiquitination in non-vascular plants is still in its infancy. For example, the polyubiquitin chain-binding subunit (PpMCB1) is regarded as one of the first reported moss ubiquitin-initiating enzymes in *P. patens*, which can bind polyubiquitin chains and help modulate the plant hormone-mediated 26S proteasomal degradation pathway [35]. By studying the evolution of the protein COP1/SPA encoded by the E3 ligase complex CUL4s, it was found that the COP1 protein maintained functional conservation in *P. patens*, rice, and *A. thaliana*, while the SPA protein showed considerable variability across species. It is speculated that this variability may be related to the number of copies of coding genes in different species [36]. A U-box E3 ubiquitin ligase, PnSAG1, was cloned from the moss *Pohlia nutans*, which grows in extreme environments on the Antarctic continent. After overexpression of the gene in *P. patens* and *A. thaliana*, it exhibited ABA treatment and salt sensitivity to stress, which indicated that PnSAG1 is a negative regulator of the ABA and salt stress signaling pathways [37]. Importantly to our work, ABI3 represents the core machinery of the ABA signal pathway, and the desiccation tolerance response of moss is mediated by ABI3, with a mutation in this gene resulting in the loss of tolerance [9,38]. However, whether other proteins interact with ABI3 and regulate ABA signaling via ubiquitination in moss has not been clear.

In this study, we found that ectopic expression of the *ScABI3* gene can reduce the ABA sensitivity of *A. thaliana* during seed germination (Figure 2). Further, we showed that a potential interaction existed between ScABI3 and ScPRT1, which was supported by in vitro pulldown and in vivo BiFC assays (Figure 3, Figure 4 and Figure 5). Moreover, we identified a representative RING ubiquitin ligase ScPRT1 from *S. caninervis*, which shares a relatively high similarity with other PRT1 proteins in other plants (Figure 1b). Belonging to the C3HC4-RING member of the RING-type E3 ligase with N-terminal rule PROTEOLYSIS1 (PRT1) (Figure 1a), it may lead to ubiquitination and degradation by recognizing the N-terminal residue of the target substrate [39]. qRT-PCR experiments confirmed that *ScABI3* and *ScPRT1* genes could be induced to express under desiccation, rehydration, and NaCl treatments, and the expression trends were relatively consistent (Figure 6B–D). In contrast, antagonistic expression patterns of *ScABI3* and *ScPRT1* were seen under ABA treatment (Figure 6A). Therefore, we hypothesize that the ABA-insensitive phenotype of *ScABI3* transgenic plants is due to the ubiquitination and degradation of ScABI3 after binding to ScPRT1.

**Figure 7:**
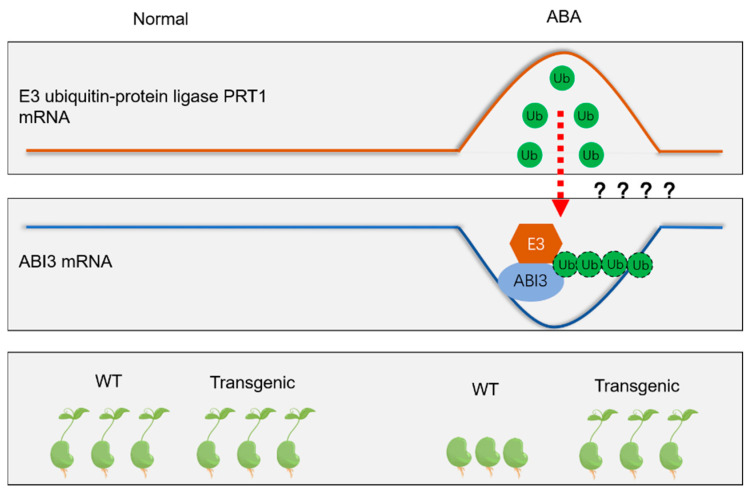
A possible model for the interaction between ScABI3 and ScPRT1 in response to ABA treatment is proposed.

Based on our data, a possible model for the interaction between ScABI3 and ScPRT1 in response to ABA treatment is proposed (Figure 7). Briefly, under normal conditions, *ScABI3* and *ScPRT1* have similar expression levels, both WT and *ScABI3* transgenic lines seeds germinate, used ABA treatment, and *ScPRT1* is highly expressed and interacts with or ubiquitinates ScABI3, resulting in the low expression of *ScABI3* and transgenic seed insensitivity to ABA. However, ubiquitination is a post-translational process of proteins degradation, the relationship between transcript levels increasing has not been proved, and the precise mechanism whereby ScPRT1 functions as an E3 ubiquitin ligase and modulates transcriptional activity of the *ScABI3* remains unclear. Further studies are needed to identify the mechanism of ScPRT1 regulation of ABA-mediated signaling by ubiquitination, which will help to clarify how protein post-translational modification regulates the ABA and drought-signaling pathways in moss.

## 5. Conclusions

In summary, we targeted a RING-type E3 ubiquitin ligase ScPRT1 and proved that ScABI3 can directly interact with ScPRT1 using Yeast two-hybrid, Pull-down, and BiFC assays in *S. caninervis*. We found that the high expression of *ScPRT1* can interfere with the transcription of *ScABI3* under ABA treatment. Our findings enrich the knowledge of the role of E3 ubiquitin ligase and provide a valuable reference for the relationship between ubiquitination modification and ABA signal transduction in desiccation tolerance moss.

## Figures and Tables

**Table 1:** Primer information for qRT-PCR.

Gene	Forward Primer	Reverse Primer	Annealing Temperature
*ScABI3*	GGTACTTCATCGTTCTGG	GTCACCGTCTAATCTCTG	60 °C
*ScPRT1*	GGAGCGCGACCAGAACAACTAC	CATCAGGTGGCCGCAGTTCATC	59 °C
*Scα-TUB*	CGGTCATTACACCGTGGGAA	CCTCTCCAGCAACAGCGAA	60 °C

## Data Availability

Not applicable.
